# Young doctors’ preferences for payment systems: the influence of gender and personality traits

**DOI:** 10.1186/s12960-015-0060-0

**Published:** 2015-08-19

**Authors:** Birgit Abelsen, Jan Abel Olsen

**Affiliations:** National Centre of Rural Medicine, Department of Community Medicine, UiT The Arctic University of Norway, N-9037 Tromsø, Norway; Department of Community Medicine, UiT The Arctic University of Norway, N-9037 Tromsø, Norway

**Keywords:** GP remuneration, Hospital payment systems, Preadapted payment preferences, Gender differences, Personality traits

## Abstract

**Objective:**

Activity-based payment contracts are common among doctors, but to what extent are they preferred? The aim of this paper is to elicit young doctors’ preferences for alternative payment systems before they have adapted to an existing system. We examine the existence of gender differences and the extent to which personality traits determine preferences.

**Methods:**

A cross-sectional survey of all final-year medical students and all interns in Norway examined the extent to which preferences for different payment systems depend on gender and personality traits. Data analysis relied on one-way ANOVA and multinomial logistic regression.

**Results:**

The current activity-based payment systems were the least preferred, both in hospitals (16.6%) and in general practice (19.7%). The contrasting alternative “fixed salary” achieved similar relative support. Approximately half preferred the hybrid alternative. When certainty associated with a payment system increased, its appeal rose for women and individuals who are less prestige-oriented, risk-tolerant or effort-tolerant. Activity-based systems were preferred among status- and income-oriented respondents.

**Conclusion:**

The vast majority of young doctors prefer payment systems that are *less* activity-based than the current contracts offered in the Norwegian health service. Recruitment and retention in less prestigious medical specialities might improve if young doctors could choose payment systems corresponding with their diverse preferences.

## Introduction

A common feature of most health systems is the extensive use of activity-based payment contracts for doctors. General practitioners (GPs) are often paid based on their productivity (fee-for-service (FFS) and/or capitation), and hospital doctors are accustomed to relatively high additional payments on top of their base-level salary as compensation for long and irregular hours [1]. While doctors themselves might argue that their behaviour is driven by professional ethics rather than by the payment mechanism, economists would claim that payment structures influence behaviour [2]. FFS provides incentives to be productive but may encourage overproduction; capitation provides incentives to serve more patients but may lead to underservice; salary provides limited incentives to be productive as the doctor is paid the same regardless of production (see, for example, [3]). More recently, the health service has been influenced by an increased interest in various pay-for-performance (“p4p”) models. However, the notion that financial incentives can improve health outcomes lacks clear evidence [4,5].

Functional payment systems are the results of negotiations and expected to be approved, if not preferred, by the members’ union (that is, the medical association). There is, however, an important element of inertia involved, as existing payment systems may reflect *past* preferences from a time when doctors were men whose norm was to work long hours while their wives took care of the domestic scene [6]. Times have changed; more women are entering the medical profession, and the new generation of doctors prefer a different work–life balance [7-11]. This is referred to as a generation and gender shift in medicine [12] or the grand gender conversion in the economic literature [13].

In addition, the doctor’s role in health care is changing. From being the curing star, the doctor is increasingly becoming an integrated team member alongside other skilled health care professionals [14]. This less autonomous role of doctors makes the use of activity- or performance-based payment systems more challenging. In addition, team membership can free doctors from irregular and long hours, as the team members to some extent substitute each other [13].

The aim of the study reported from here was to elicit young doctors’ preferences for different payment systems *before* they adapt their preferences to an existing system. More specifically, we examine (a) the existence of gender differences in preferences for payment systems and (b) the extent to which personality traits determine preferences for a payment system. Finally, we discuss the extent to which policy makers should respond to young doctors’ payment system preferences. The study provides new insights for analysing professional labour markets, in which heterogeneity in career interests is acknowledged.

### Study context

The Norwegian payment systems for doctors are quite similar to those of other OECD countries [1]. On the primary health care level, the GPs act as gatekeepers, that is, patients require a GP’s referral to access speciality care. A patient-list system gives all citizens the right to sign up on a GP list. In 2014, the average number of patients on such lists was 1150 .The vast majority of Norwegian GPs have private practices and receive approximately one third of their incomes based on capitation (flat rate per patient) paid by the municipalities and the remaining two thirds based on FFS. The FFS scheme is a mix of a fixed fee per consultation paid by patients and variable fees paid by the government depending on the following: the duration of the consultation, whether certain types of examinations and laboratory tests are initiated and whether the doctor is a specialist in general medicine [15]. The payment system for doctors in hospitals is salary-based and offers strong incentives for working irregular hours. On average, hospital doctors make an additional 23% of their income from irregular hours and overtime work (19% among women, 26% among men) [16]. The hospitals operate on public budgets, and hospital doctors are employed in local health authorities covered by collective agreements. Wage-setting for employees covered by collective agreements takes place at two levels: national and firm. At the national level, wage regulations, working hours, working conditions, pensions and medical benefits are negotiated. The firm-level negotiations determine possible local adjustments and additions to the collective agreements [17].

Female doctors work on average 4 h less per week than do male doctors [18]. Over the last decade, the number of practising physicians has increased by 32%, while the proportion of female doctors has increased from 36% to 46% [19]. Due to the activity-based payment structure, the GPs’ salaries are currently less predictable than hospital doctors’ salaries. The GPs’ share of predictable income could be seen as comprising one third of total income, while the similar hospital doctors’ share comprise 77% of total income. However, unlike many other OECD countries [1], the average payment levels are higher among Norwegian GPs than among hospital doctors [20].

The Norwegian health care system is organized within two sectors. Municipalities are responsible for primary care, while specialist care is the responsibility of the state (administered by four regional health authorities). The two sectors have different funding mechanisms, as well as different administrative, political and professional cultures [21]. In the past few years, policy reforms have been implemented to improve coordination and integration between provider levels to control rising costs.

### Gender and personality traits

Several studies have shown that male physicians are more *externally* motivated in their career choices by aspects of income, status and the opportunity to implement technical activities, whereas females are more *intrinsically* motivated by humanist and altruistic aspects [22,23]. Given that women tend to prefer more predictable income and fewer financial incentives than men [24], we would expect female physicians to be less happy with the current activity-based payment systems in general practice. Given the increasing evidence that women have stronger aversions than men to working long irregular hours [13], we expect them to be even more reluctant to the current payment system in hospitals that disproportionately rewards doctors who are prepared to work accordingly. Furthermore, we expect a payment system that involves disproportionate compensations for sacrificing leisure time to conflict with the preferred work–life balance of young doctors who have a family.

Based on previous research [20,24,25], we further expect the current payment systems to be preferred by those having personality traits involving prestige-orientation and status-seeking and by those who are risk-tolerant, effort-tolerant and income-focused.

An early study on women in medicine showed that women had chosen specialities that tended to have lower *prestige* and lower income [26]. It was, however, unclear whether certain specialities carry less prestige because they have a high number of women. Several studies have shown that male physicians are more likely to choose surgery as a speciality, whereas female physicians tend to prefer general practice [27-30].

It is well known that the medical community (both medical students *and* experienced physicians) assign different prestige to medical specialities [31-36]. A *prestige hierarchy* has been developed to rank specialities and subspecialities on a scale from 1 (geriatrics) to 22 (neurosurgery) [31,32,37]. General practice is ranked near the bottom of this list (index 4). Studies on women in medicine show that women compared to men tend to choose specialities that have lower *prestige* and lower income [11,26,27,38,39].

Based on the above, we expect the current activity-based payment system in general practice to be relatively *less* popular among women and risk-averse doctors. On the contrary, we expect this system to be *more* popular among income-oriented and effort-tolerant doctors. The payment system in hospitals involving disproportionate rewards for long and irregular hours is also expected to be relatively *less* popular among women and particularly so among those having a family. Doctors who are prestige- and status-oriented are expected to approve of a system that rewards their inclinations to work long and irregular hours, something they consider to be required for becoming a specialist at an early age.

## Methods

A cross-sectional study was conducted at the end of 2010 among all last-year medical students and all interns in Norway (*n* = 1562). Contact information was provided by the four medical faculties and the organizers of internships (local health authorities and county governor offices). The information letter included a web link to an online questionnaire. Two reminders were mailed. The survey was reported to the Data Protection Official for Research in Norway in accordance with notification requirements.

Two questions were asked about respondents’ *preadapted* preferences for payment systems (prior to their entry into regular medical work). The first question was, “Which payment system would you prefer if you were to work as a GP and had a free choice?” The response options were (a) fixed salary, (b) activity-based income (that is, current system), (c) a combination in which a percentage is fixed and the rest is activity based and (d) don’t know. Those who opted for the combined system were asked a follow-up question including an open space to fill in their preferred fixed-salary percentage.

The subsequent question was, “Which payment system would you prefer if you were to work as a hospital doctor and had a free choice?” The response options were 1) current payment system (relatively low base salary but possibility for relatively high total income depending on additional irregular working hours), (b) a shift towards a higher base salary and less weight on additional pay from irregular working hours, (c) fixed salary with normal working hours (8 a.m. to 4 p.m.) and (d) don’t know. Prior to these questions, respondents were informed (or reminded) about the nature of the current payment systems in hospitals and general practice.

While not explicitly mentioned in the wording of the questions, it was implicit that the average payment level would be the same across payment systems. All respondents were expected to understand that by definition the payment *range* would be larger under variable payment systems.

The answers to these two questions were expected to depend on differences in respondent characteristics such as gender and family situation (marital status and having children), as well as differences in personality traits such as prestige orientation, risk attitude and orientation towards high income, status and work efforts.

### Prestige orientation

From a list of 30 different specialities and subspecialities [40], subjects were asked to indicate which ones they were considering. In the analyses, the specialities were ranked in accordance with their prestige index [37], from which the mean index of the alternatives indicated was calculated. The mean value, ranging from 1.5 to 22, was used as a measure of respondents’ *prestige orientation*: the higher the value, the more prestige-oriented is the respondent.

### Risk attitude

Respondents’ *risk attitude* was measured by six risk-related items from the Jackson personality inventory-revised [41], adapted and validated by Pearson et al. [42]:

1. I enjoy taking risks
2. I try to avoid situations that have uncertain outcomes
3. Taking risks does not bother me if gains involved are high
4. I consider security an important element in every aspect of my life
5. People have told me that I seem to enjoy taking chances
6. I rarely, if ever, take risks when there is another alternative.

These items have been used in several studies of medical decision making [43-46]. The respondent scored all items on a Likert scale from strong disagreement (1) to strong agreement (6). The average scores of these six items made an index ranging from 1 (very risk-averse) to 5.3 (very risk-seeking). In the construction of the risk attitude index, statements 2, 4 and 6 were reversely recorded. The higher this index, the more *risk-prone*.

### Income, status and work-pace tolerance

Preferences for high income, status and work-pace tolerance were measured using statements with which respondents were asked to state their level of agreement on a six-point Likert scale ranging from strong disagreement (1) to strong agreement (6). The wordings of these statements were, “It is important for me to have a high income”, “I want a job that gives me a high status among other doctors” and “I am happy with a high work pace”. For analysis purposes, the ordinal values 1 to 6 were treated as an interval scale, assuming it to be a reasonable approximation [47].

### Data analyses

The data were initially analysed by frequency counts, contingency tables, means and medians. A chi-square test was used to establish whether there were significant gender differences in the preferred payments systems. Statistical tests with *P* values less than 0.05 are interpreted as a sign of statistically significant results. One-way ANOVA was used to test for differences in respondent characteristics among the groups giving different answers to the two main questions analysed. Multinomial logistic regression analysis [48,49] was then used to profile respondents who preferred another payment system to the current system in hospital and general practices. SPSS version 22.0 was used to perform the statistical analyses.

## Results

A total of 831 persons (53%) responded. The gender balance was identical to that in the invited sample. We have no reason to anticipate biases in the sample regarding gender, age and place of living. However, like in surveys more generally, we cannot preclude the possibility of self-selection related to some “hidden” preferences of relevance to the issues under consideration.

Table 1 provides respondent characteristics: mean age of 28.4 years, 59% female and 54% having a family (defined as married/cohabiting (53%) and/or having children (21%)).

**Table 1:** Respondent characteristics, total sample

			***n***
Age, mean (range)	28.4 (23–53)		818
Gender, male	41%		830
Married/cohabiting	53%		830
Have children	21%		829
Family, that is, married/cohabiting and/or have children	54%		829
*Personality traits*	Mean (range)	Median	
Prestige score	9.5 (1.5–22)	9	804
Risk-prone index	2.9 (1–5.3)	2.8	816
Status-oriented	2.5 (1–6)	2	830
Income-oriented	4.2 (1–6)	4	828
Effort-tolerant	4.1 (1–6)	4	828

The mean (and median) prestige score is quite low and reflects that the majority (55%) of respondents had indicated general practice (prestige index 4) as one alternative speciality. On average, the respondent indicated 3.2 specialities. The risk-prone index suggests a normal distribution (17% scored 1 standard deviation (SD) below the mean, while 18% scored 1 SD above the mean). The average respondent did not seem very concerned about achieving a high status among other doctors. The average respondent did, however, appear to be more oriented towards achieving a high income and to tolerate a high work pace.

### Is there a gender difference in payment system preferences?

Tables 2 and 3 present the three different payment systems we investigated, in hospitals and general practice, respectively, in accordance with their degrees of income predictability or certainty. While fixed salary is the payment system that by definition provides a predictable income, the current payment systems involve uncertainty. The “combined systems” were presented as compromise alternatives, involving a higher proportion of predictable fixed income.

**Table 2:** Gender differences in preferred payment systems in hospitals

	**Current (%)**	**Combined (%)**	**Fixed salary (%)**	**Don’t know (%)**	**Total (%)**	***n***	**Chi square ** ***P*** ** value**
Total	16.6	48.6	19.7	15.1	100	829	
Male	21.0	50.6	17.2	11.2	100	338	0.002
Female	13.5	47.3	21.4	17.8	100	490

**Table 3:** Gender differences in preferred payment systems in general practice

	**Current (activity-based) (%)**	**Combined (%)**	**Fixed salary (%)**	**Don’t know (%)**	**Total (%)**	***n***	**Chi square ** ***P*** ** value**
Total	19.7	47.9	20.4	12.1	100	829	
Men	27.7	50.1	14.2	8.0	100	339	<0.001
Female	14.1	46.4	24.7	14.7	100	489	

Interestingly, the current payment systems were the least preferred, both in hospitals (16.6%) and in general practice (19.7%). The most preferred payment system in hospitals (48.6%) would be one with a higher proportion of fixed income (that is, fewer incentives for working irregular hours), while the most preferred payment system (47.9%) in general practice was the one combining activity-based payment with fixed salary. Those who opted for such a blended system in general practice stated a preferred percentage of fixed salary, which ranged from 25% to 80% (mean 57.6%, median 50%). The “fixed salary” option achieved similar relative support both in hospital and in general practice.

The results in Tables 2 and 3 indicate that young female and male doctors have significantly different payment system preferences: Women prefer even less income variability than men. This difference is particularly apparent in their preferences for payment systems in general practice.

### Who are attracted to which payment system in hospitals?

Table 4 reports the binary one-way ANOVA analyses comparing the mean values of various respondent characteristics attracted by the different hospital payment systems. There are statistically significant differences related to gender, family situation, prestige orientation, risk attitude, income orientation and effort tolerance. These binary analyses confirm that the more certainty there is in the payment system, the more it appeals to women and to respondents with a family. That is also the case among those who are less prestige-oriented, less risk-tolerant and less effort-tolerant.

**Table 4:** Differences in payment system preferences in hospitals by doctor characteristics

	**Current**	**Combined system**	**Fixed salary**	**Don’t know**	***n***	
**One-way ANOVA analyses**	**Means**					***P*** ** value**
Gender (1 = male)	0.52	0.42	0.36	0.30	828	0.002
Family (1 = yes)	0.43	0.57	0.61	0.50	828	0.009
Prestige score	10.56	9.90	8.24	8.54	802	<0.001
Risk-prone index	3.09	2.91	2.82	2.83	814	0.034
Status-oriented	2.64	2.58	2.38	2.46	828	0.244
Income-oriented	3.99	4.39	4.08	3.98	826	<0.001
Effort-tolerant	4.44	4.25	3.70	3.85	826	<0.001

Table 5 shows the result of a multinomial logistic regression analysis elucidating the profiles of respondents who preferred a different payment system in hospitals to the current one. The analysis only includes the statistically significant respondent characteristics from Table 4. Compared with those preferring the current payment system, those preferring a *combined system* are more likely to have a family and be income-oriented. Those preferring *fixed salary* are more likely to be women and have a family. Furthermore, they are much less tolerant of a high work pace, are less concerned about prestige and seem to be slightly more income-oriented.

**Table 5:** Multinomial logistic regression analysis of preferred hospital remuneration system

	**Odds ratio (95% CI)** ^**a**^			
	**Combined system ** ***n*** ** = 386**	**Fixed salary ** ***n*** ** = 149**	**Don’t know ** ***n*** ** = 117**	**Current ** ***n*** ** = 131**
Gender (0 = female, 1 = male)	0.76 (0.50–1.16)	0.58 (0.34–0.97)*	0.44 (0.25–0.77)**	Reference category
Family (0 = no, 1 = yes)	1.70 (1.13–2.57)*	1.72 (1.05–2.84)*	1.11 (0.66–1.88)	
Prestige score	0.98 (0.94–1.03)	0.93 (0.87–0.98)*	0.93 (0.87–0.99)*	
Risk-prone index	0.98 (0.94–1.02)	0.99 (0.94–1.05)	1.00 (0.94–1.05)	
Status-oriented	Not included	Not included	Not included	
Income-oriented	1.43 (1.19–1.72)***	1.25 (1.01–1.55)*	1.14 (0.91–1.43)	
Effort-tolerant	0.84 (0.69–1.02)	0.61 (0.48–0.76)***	0.67 (0.52–0.85)**	
Model fit information	Cox and Snell *R* ^2^ = 0.113, Nagelkerke *R* ^2^ = 0.123			

^a^Statistically significant: *: *P* < 0.05; **: *P* < 0.01; ***: *P* < 0.001.

Interestingly, the two tables indicate that the group that responded “don’t know” to which hospital payment system it preferred has similar respondent characteristics and personality traits to those of the “fixed salary” group.

### Who are attracted to which payment system in general practice?

Table 6 reports the binary one-way ANOVA analyses comparing the mean values of various characteristics of respondents attracted by the different payment systems in *general practice*. There are statistically significant differences between the groups related to gender, risk attitude, status, income and effort. Again, the analyses confirm that the more certainty there is in the payment system, the more it appeals to women and those with low risk tolerance. Those most concerned with status and having a high income preferred the current activity-based payment system; the same was true for those with tolerance for a high work pace.

**Table 6:** Differences in payment system preferences in general practice by doctor characteristics

	**Current (activity based)**	**Combined**	**Fixed salary**	**Don’t know**	***n***	
**One-way ANOVA analyses**	**Means**					***P*** ** value**
Gender (1 = male)	0.58	0.43	0.28	0.27	828	<0.001
Family (1 = yes)	0.53	0.53	0.54	0.59	828	0.937
Prestige score	9.67	9.78	8.95	9.43	789	0.129
Risk-prone index	3.07	2.95	2.69	2.84	814	<0.001
Status-oriented	2.85	2.56	2.29	2.42	828	0.001
Income-oriented	4.60	4.25	3.92	3.88	826	<0.001
Effort-tolerant	4.52	4.13	3.80	3.93	826	<0.001

Table 7 shows the result of a multinomial logistic regression analysis elucidating the profiles of respondents who preferred a different payment system in general practice to the current one. The analysis only includes the statistically significant characteristics from Table 2 as independent variables. Compared to those preferring the current payment system, those preferring the other systems are likely to be women, less income-oriented and less effort-tolerant.

**Table 7:** Multinomial logistic regression analysis of preferred general practice remuneration system

	**Odds ratio (95% CI)** ^**a**^			
	**Combined ** ***n*** ** = 390**	**Salary ** ***n*** ** = 163**	**Don’t know ** ***n*** ** = 96**	**Current (activity-based) ** ***n*** ** = 160**
Gender (0 = female, 1 = male)	0.59 (0.40–0.88)**	0.39 (0.24–0.63)***	0.34 (0.19–0.61)***	Reference category
Family (0 = no, 1 = yes)	Not included	Not included	Not included	
Prestige score	Not included	Not included	Not included	
Risk-prone index	1.01 (0.97–1.02)	0.97 (0.92–1.02)	1.00 (0.95–1.06)	
Status-oriented	0.96 (0.82–1.11)	0.90 (0.75–1.10)	0.99 (0.80–1.24)	
Income-oriented	0.79 (0.65–0.95)*	0.65 (0.51–0.81)***	0.60 (0.46–0.78)***	
Effort-tolerant	0.74 (0.62–0.89)**	0.65 (0.52–0.80)***	0.68 (0.53–0.87)**	
Model fit information	Cox and Snell *R* ^2^ = 0.114, Nagelkerke *R* ^2^ = 0.124			

^a^Statistically significant: *: *P* < 0.05; **: *P* < 0.01; ***: *P* < 0.001.

Similar to Tables 4 and 5, when looking at the characteristics of the “don’t know” group in Tables 6 and 7, respondents have a profile quite similar to those who prefer a fixed salary.

## Discussion

This study supports the views expressed among young doctors in many countries that an activity-based payment system in general practice and a hospital payment system that disproportionately rewards long and irregular working hours do not correspond with their preferences. Only 16.6% of those entering the medical profession in Norway prefer the current payment system in hospitals (low base salary and high compensation for long and irregular working hours). In general practice, the current payment system involving a blend of fee-for-service and capitation was preferred by 19.7%.

Interestingly, the even lower support for the current payment system in hospitals suggests that a system with an *increasing* marginal compensation for long hours is even less popular than one with *constant* marginal compensation for increased hours, like the activity-based system used for GPs. While increasing marginal rewards is an efficient way of inducing labour supply (in that the substitution effect trumps the income effect), it might still be considered unpopular because it puts further pressure to divert from a work–life balance preferred by the new generation of doctors, in particular women and those having a family.

The results clearly show that young doctors prefer payment systems that provide a more predictable income. This is emphasized by the fact that the “fixed salary” option received slightly higher supports than the current systems. The most preferred payment models were hybrids between the current systems and fixed salary. The popularity of these hybrid models might reflect a perception that they involve less uncertainty and pressure, while the total payment remains the same.

As expected, there are significant gender differences in payment system preferences. Women have a stronger preference for a more predictable income, particularly in general practice. Only half as many women compared to men prefer the current GP remuneration system (14.1% vs 27.7%). Having a family was a significant determinant for explaining variations in preferences for payment systems in hospitals. Briscoe [50] points out that doctors who are also primary caregivers saddle with responsibilities associated with both their families and their patients. Situations that require flexibility to decide when and for how long to engage in work activity are likely to arise in both spheres.

This survey provides support for the idea that different payment systems attract people with different personality traits. Young doctors with an affinity for prestigious medical specialities are those who are most in favour of the current payment system in hospitals. Success within a prestigious speciality is associated with long working hours, which may explain why *prestige-oriented* young physicians to a lesser extent prefer the fixed salary option, described as working normal hours from 8 a.m. to 4 p.m.

While differences in prestige orientation did not affect preferences for GP payment systems, so did differences in *status orientation*. The more status-oriented you are, the more income variability in general practice you accept. However, status orientation did not explain differences in preferences for hospital payment systems.

As expected, risk-prone respondents were more prepared to accept income variability. The income-oriented respondents showed a significantly stronger affinity for the current activity-based payment system in general practice. Finally, the variable “effort-tolerant” was significant for explaining preference variations for payment systems in both hospitals and general practice. The more you dislike having to work at a high pace, the more you would like a payment system with a predictable income.

The aversion towards the current payment systems, as measured by the support for the contrasting alternative (fixed salary), appears to be strongest in general practice. The reason might be that the current activity-based system for GPs is fundamentally different from the current salary-based system in hospitals in terms of income predictability and certainty. The current activity-based system for GPs results in poorer earnings if you are unable to maintain activity at a high level. This element of individual risk accentuates by the fact that GPs themselves are responsible for arranging their own pensions and sick-leave insurance. Hospital doctors, in contrast, have a base salary and are included in the public social security system. Thus, becoming a GP appears to involve more of a commitment than becoming a hospital doctor.

One might argue that it does not really matter what young physician initially think about the payment systems because they will adapt to the systems as soon as they become part of them. However, cross-sectional studies among experienced Norwegian GPs show that one in two prefers a different payment system from their current one and that this proportion has increased significantly over the last couple of years [51,52]. Rather than their current activity-based system, one in three would prefer a fixed salary. The study shows that physicians do not necessarily learn to adapt to the existing payment system and that there is a significant mismatch among those who have had many years to adapt. Similar studies among experienced Norwegian hospital doctors about their views on the existing payment system have not been found. This calls for further research. Halvorsen et al. [51] point out that the ability to focus on patients’ needs may also play a significant role in the formation of payment system preferences.

### Should young doctors’ payment system preferences be taken into account?

An optimal payment system in the health sector is that which best contributes to improving patients’ health, not the utility (or income) of doctors. If it in addition keeps the costs down, it certainly would be preferred from a political point of view. In principle, one may therefore find it neither surprising nor worrisome that young doctors display some degree of dissatisfaction with the current payment systems. The result might rather suggest that policy makers are adequately protecting patients’ interests by “imposing” high effort on doctors and monitoring their actions. However, if doctors’ dissatisfaction reduces their motivation, this may have negative consequences for the quality of care provided and possibly also for recruitment and retention. In negotiating proper payment systems, health authorities should not only care about physicians’ productivity but also the impact on continuity of patient care. Contract type is known to be one of many factors that influence recruitment and retention [53,54]. There is also evidence suggesting that specific initiatives reducing workload as such are effective [55,56].

Designing payment systems to satisfy effort-intolerant workers might be seen as a risk of productivity reduction. Theories on economic incentives suggest that a lack of activity-based payment will lead to shirking [57], meaning that unless incentives induce people to work, they are expected to devote minimum effort. There are, however, other theories about professions and public service motivation, as well as empirical evidence indicating that behaviour and performance among health professionals are determined by aspects beyond financial incentives [58-60]. Green [60] finds that physicians are intrinsically motivated to provide high quality care and warns that relying exclusively on extrinsic incentives is detrimental to the quality of care and costly for the health care industry.

The fact that young female doctors have significantly different preferences for payment systems than males also represents a policy challenge given the new gender balance in medicine. There are no obvious reasons why policy makers would wish to maintain payment systems that might create gender imbalance in certain types of sectors or specialities. To the extent that recruitment and retention of doctors, as well as more integrated care, are important policy goals, our findings suggest that health authorities may do well in supplementing the “prehistoric” remuneration systems for doctors by offering more payment system diversity. This is in line with revealed preferences and developments in other countries. The young generation of physicians in France demands a larger choice of payment schemes. Choice of payment system exists in the US where physicians can choose between capitation and FFS payments. Similarly, in the Canadian Province of Quebec, specialists can choose between FFS and a mixed payment scheme (part FFS, part capitation) [61].

Allard et al. [61] examine the consequences of allowing physicians to self-select into FFS or capitation payment schemes in a setting where GPs act as gatekeepers to specialized care. If the main concern is to reduce the specialized care costs, it is optimal from the regulator’s perspective to pay all GPs on a FFS basis and not let them choose the payment scheme. If the main concern is quality of health outcome, the answer depends on the GP’s ability. With mainly high-ability GPs, self-selection of a payment is optimal. On the opposite, with mainly low-ability GPs, capitation is optimal.

## Conclusion

The vast majority of young doctors prefer payment systems that are *less* activity-based and puts less pressure on working long and irregular hours than do the current contracts offered in the Norwegian health service. There are reasons to expect recruitment and retention might improve in the less prestigious medical specialities if the new generation of doctors had the opportunity to choose a payment system that corresponds with their diverse preferences.
